# Potential Threats Posed by New or Emerging Marine Biotoxins in UK Waters and Examination of Detection Methodology Used in Their Control: Brevetoxins

**DOI:** 10.3390/md13031224

**Published:** 2015-03-12

**Authors:** Andrew D. Turner, Cowan Higgins, Keith Davidson, Andrea Veszelovszki, Daniel Payne, James Hungerford, Wendy Higman

**Affiliations:** 1 Centre for Environment Fisheries and Aquaculture Science (Cefas), Barrack Road, The Nothe, Weymouth, Dorset DT4 8UB, UK; E-Mails: Daniel.payne@cefas.co.uk (D.P.); Wendy.higman@cefas.co.uk (W.H.); 2 Agri-food and Biosciences Institute (AFBI), Newforge Lane, Belfast BT9 5PX, UK; E-Mail: cowan.higgins@afbini.gov.uk; 3 Scottish Association for Marine Science (SAMS), Oban, Argyll PA37 1QA, UK; E-Mails: keith.davidson@sams.ac.uk (K.D.); Andrea.veszelovszki@sams.ac.uk (A.V.); 4 University of Surrey, School of Biosciences and Medicine, Guildford, Surrey GU2 7TE, UK; 5 United States Food and Drug Administration (USFDA), 22201 23rd Dr, S.E., Bothell, WA 98021, USA; E-Mail: James.hungerford@fda.hhs.gov

**Keywords:** *Karenia*, brevetoxin, phytoplankton, shellfish

## Abstract

Regular occurrence of brevetoxin-producing toxic phytoplankton in commercial shellfishery areas poses a significant risk to shellfish consumer health. Brevetoxins and their causative toxic phytoplankton are more limited in their global distribution than most marine toxins impacting commercial shellfisheries. On the other hand, trends in climate change could conceivably lead to increased risk posed by these toxins in UK waters. A request was made by UK food safety authorities to examine these toxins more closely to aid possible management strategies, should they pose a threat in the future. At the time of writing, brevetoxins have been detected in the Gulf of Mexico, the Southeast US coast and in New Zealand waters, where regulatory levels for brevetoxins in shellfish have existed for some time. This paper reviews evidence concerning the prevalence of brevetoxins and brevetoxin-producing phytoplankton in the UK, together with testing methodologies. Chemical, biological and biomolecular methods are reviewed, including recommendations for further work to enable effective testing. Although the focus here is on the UK, from a strategic standpoint many of the topics discussed will also be of interest in other parts of the world since new and emerging marine biotoxins are of global concern.

## 1. Introduction

**Figure 1:**
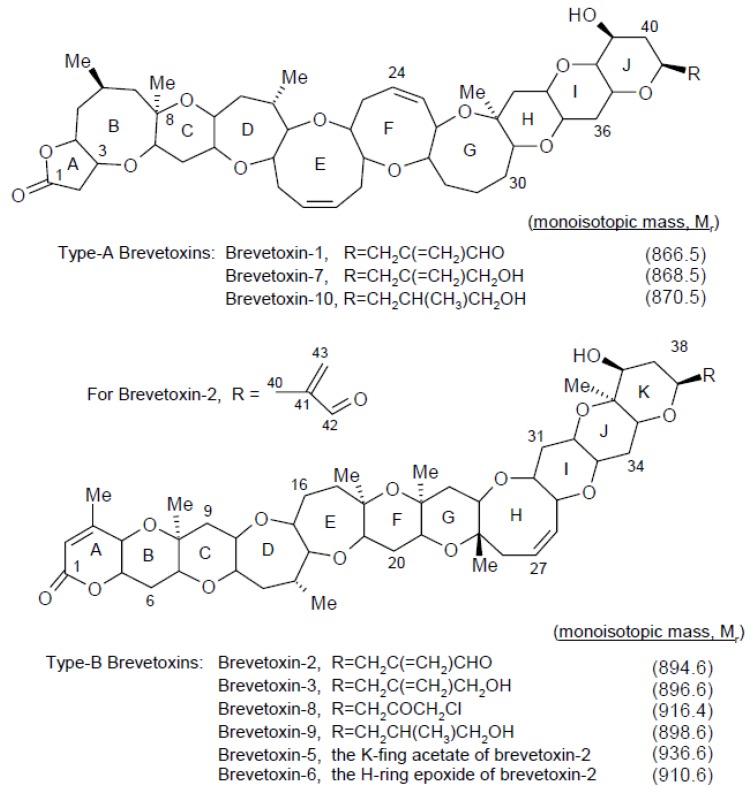
Brevetoxin chemical structures (adapted from [2]).

**Figure 2:**
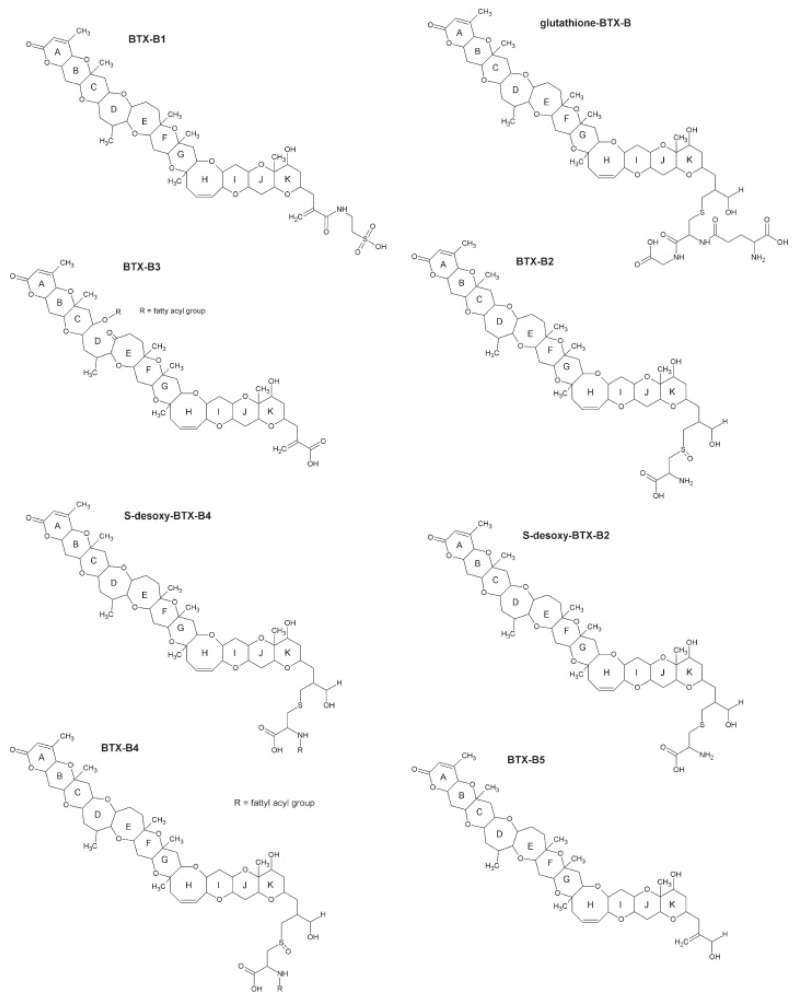
Brevetoxin metabolite chemical structures (Figure modified from [3,4]).

The brevetoxins (BTXs or PbTxs) are a large family of cyclic polyether compounds and their metabolites. The initial, phytoplankton forms are primarily produced by the dinoflagellate genus *Karenia*, most notably the species *K. brevis* (also known as *Gymnodinium breve* or *Ptychodiscus breve*) [1]. It is now known that toxins produced by such algae are rapidly metabolized in many animals including shellfish [2], and so a variety of very different toxin profiles are responsible for toxicity in fish, shellfish, marine mammals, birds and humans. Figure 1 shows the structures of the algal forms, while Figure 2 illustrates some of the most important metabolites.

Blooms of the causative algae result in both food safety and environmental damage, being responsible for mass mortalities of fish and other marine organisms. BTXs are neurotoxins with high affinity to the receptor sites of voltage gate sodium channels and can affect mammalian cortical synaptosomes and neuromuscular preparations [5]. These effects are associated with significant membrane depolarization. Respiratory effects are also due to the interaction of the toxins with the voltage gated sodium channels on nerve cell membranes [6]. Humans may be affected from BTX exposure through consumption of contaminated shellfish, termed neurotoxic or neurologic shellfish poisoning (NSP), or from exposure to aerosols on or near to marine waters where algal blooms have developed.

Following intraperitoneal (*i.p.*) injection into mice, the BTX-group toxins cause depression of respiratory and cardiac function, muscular contractions, twitching and leaping, paralysis and death. The European Food Standards Agency (EFSA) report indicates that there is limited toxicity data available on the BTX group and their metabolites. Signs of intoxication following *i.p.* injection in mice have been described for BTX-B2, BTX-3 and S-deoxy-BTX-B2 with lethal doses inducing immobility after 15 min followed by respiratory paralysis and death. At sublethal doses, fast abdominal breathing immediately following injection was noted. Subsequent paralysis occurred with movement regained after 3–5 h. Following intravenous (*i.v.*) injection, toxic signs and death were immediate. Oral toxicity data for BTX-2 indicates a LD_50_ value of 6600 mg/kg b.w. whilst for BTX-3 a figure of 520 mg/kg b.w. is reported [7]. The authors report that the difference in values may be due to differences in the absorption rate of the toxin analogues. Recent studies using mice have also shown large variations in the subsequent tissue distribution of different metabolites, with some being found in liver, spleen, and lungs, others predominantly in the kidney, and others more widely distributed [8]. Several studies have indicated the potential for BTXs to induce chromosomal damage. BTX-2 induced chromosomal aberrations in hamster ovary cells [9] and DNA damage was found in Jurkat E6-1 cells following exposure to BTX-2, BTX-3 and BTX-6 [10]. EFSA reported that *i.p.* toxicity for BTX-2, BTX-3, BTX-B2 and *S*-deoxy-BTX-B2 are similar but there was insufficient data on which to evaluate the relative potencies of other analogues. It was noted that the oral toxicity of BTX-3 appears to be a factor of 10 higher than that of BTX-2 [11]. In addition to the parent toxins and related products present in the algae, an antagonist, brevenal has also been discovered, a compound which inhibits the toxicity of BTXs through competitive replacement of BTXs at the sodium channel binding site [12,13].

Symptoms of NSP following human exposure to brevetoxin-contaminated shellfish include both gastrointestinal and neurological. These are apparent within 30 min to 3 h after consumption exposure and may last for several days. Reports of deaths or chronic conditions are not recorded [3,11]. BTXs are thought to be more toxic to humans following inhalation than following shellfish consumption [4,14]. Aerosols are formed as the dinoflagellate cells are broken down, releasing the toxins into the water and subsequently into the atmosphere under appropriate atmospheric conditions. Inhalation can cause irritation in the eyes, nose and throats and may cause respiratory distress [15] particular in asthma sufferers [16]. Symptoms appear to be reversible when subjects are removed from the area of exposure, although there is recent evidence for hyper-responsiveness and lung inflammation following repeated exposure to aerosols of PbTx-3 [17].

Large documented outbreaks of NSP have occurred in New Zealand during 1992–1993 and periodically in the Gulf of Mexico and along the east coast of the USA [5]. Large differences between shellfish species have been reported for the accumulation and reduction in concentration of toxins and their associated metabolites. There is wide variability in toxin depuration rates between the different BTXs and related metabolic products, given large differences between the toxins in terms of polarity and hydrophobicity [18]. In some cases shellfish such as oysters have been found to remain toxic more than 70 days after the dissipation of the source algal bloom [19] and there are reports of depuration taking up to nearly one year post bloom [5]. In other studies, clearance rates were determined for four different species of bivalves, with results showing significant differences between scallops, clams and oysters [20] and in four species of invertebrates including a sponge, a tunicate and a clam [21]. In other studies oysters have been shown to exhibit rapid accumulation and reduction, thought to relate to their high filtration rates, where as other species such as clams show longer depuration times [13]. There is a notable absence of information regarding the effects of product processing on the levels of brevetoxins in shellfish [11].

## 2. Potential Brevetoxin-Producing Phytoplankton Threats for UK Waters

The species of *Karenia* most frequently linked with NSP through its production of BTXs, *K. brevis* [22] has not been recorded in UK waters. However, another species of the genus, *Karenia mikimotoi*, is common in UK waters and has been responsible for the mortality of farmed fish [23,24]. *K. mikimotoi* has been demonstrated to produce gymnocins [25] although these have been observed only to be weakly toxic to fish [24]. The organism is not thought to be harmful to humans, with fish mortality being caused by a combination of the ichthyotoxins and the deoxygenation (associated with high biomass blooms).

Historically, *K. brevis* was considered to be restricted to the Gulf of Mexico and the east coast of Florida, where it is endemic.Human cases of NSP were unexpectedly reported in the summer of 1992–1993 on the NE coast of New Zealand. These were associated with a previously unknown dinoflagellate named *K. cf brevis.* This organism is similar to other *Karenia* species (*K. mikimotoi* and *K. brevis*) in morphology, and produced “brevetoxin-like” lipid soluble toxins [26]. Phytoplankton growth and species composition is often controlled by temperature, with upper and lower temperature barriers to growth [27] Summer sea surface temperatures in this region of New Zealand are typically ~21 °C and hence exceed the average levels found within most of the UK coastline.

Subsequently, a species named *Karenia brevisulcata* was found in New Zealand waters. In 1998 it caused a severe harmful algal bloom (HAB) incident in the central and southern east coast of the North Island which devastated all marine life in Wellington Harbour [28]. Over 500 cases of human respiratory distress were reported during this event, although no food poisoning associated with the event was recorded. Symptoms included dry cough, severe sore throat, rhinorrhoea, skin and eye irritations, severe headaches and facial sunburn sensations. The respiratory distress was attributed to exposure to seawater and aerosols and resembled that caused by aerosolised BTXs [29]. A range of novel toxins were identified in bulk cultures of algae, although no brevetoxins were identified. In particular a total of 6 water-soluble brevisulcatic acids (BSX) were isolated and structurally elucidated from the acid fractions of algal cultures, showing similar structures to the brevetoxins [30,31]. Furthermore, 4 lipid-soluble Brevisculcenals (KBT) were isolated from the neutral fraction of cultures and purified toxins were produced. KBT have been found to be cytotoxic against mouse leukemia cells, with mouse lethalities of 40 µg/kg and 32 µg/kg for KBT-F and KBT-G respectively [29,32]. No blooms of *K. brevisulcata* have since been reported, so it is difficult to speculate on the cause of this outbreak. It was noted that the water was unusually warm and stratified at the time of the bloom. Given that water temperatures near Wellington (~18 °C in in summer) are closer to those in the UK, blooms of novel *Karenia* species which could be potentially harmful to humans cannot be discounted in UK waters. As part of this review, a range of UK and European agencies, research establishments and universities were contacted regarding any further potential occurrences of new/emerging toxin producing species. In response to this contact a tentative identification of *Karenia papilionacea* was reported by Marine Scotland Science (MSS) [33]. This organism may produce BTXs [34]. The observation highlights the possibility of new *Karenia* species appearing in the UK with the potential for introduction of brevetoxins into the food chain and subsequent health implications for the shellfish consumer. It has previously been identified in New Zealand, but [34] noted that *K. papilionacea* like species have also been described from Spain [35]. Also a species described as *K. brevis* from Japan [36] is more similar to *K. papilionacea* than to *K. brevis*.

To date, no other phytoplankton species detected in UK waters have been reported to contain brevetoxins. However, in the future a number of factors could conceivably contribute to the establishment or bloom development of brevetoxin-producing phytoplankton in the UK. Possible factors include ballast water transfer, climate change and changes to the anthropogenic nutrient input and nutrient ratios. There is little evidence that the latter two factors are important in HAB development in UK waters [37,38].

Transfer of HAB species through the movement of water in shipping vessels has been known for some time [27]. Ballast water transfer is of concern in the UK with [39] finding Scottish coastal waters to be “open” to ballast water and sediment introductions of toxic and benign species of phytoplankton, including both immigrant and indigenous species. They found this exposure to be of concern, given the significant increase in harmful and novel species blooms recorded in European waters. However, whilst a number of studies relating to ballast water transfer have been published, very few have focused on the transportation of *Karenia* species. In the UK, [40] and [41] reported surveys of 127 vessels arriving at Scottish ports and 76 vessels at English and Welsh ports, none of these included brevetoxin-producing organisms. However, these studies do demonstrate the potential for phytoplankton transportation in ballast water and sediment, with [42] demonstrating that, even with transoceanic ballast water exchange that is intended to prevent algal translocations, ballast water remains a potential vector for HAB dispersal.

An International Council for the Exploration of the Sea (ICES) workshop on “harmful phytoplankton that could potentially be transported or introduced by ballast waters” was held in October 2012 [43]. The report from the workshop concludes that there was not a lack of information regarding the species of phytoplankton capable of surviving in ballast tanks and the associated sediments. The report did, however, generate a list of harmful phytoplankton species that could potentially be transported in ballast water that included representatives of the *Karenia* genera.

The role of climate change in governing the distribution of HAB species must also be considered [27,44,45]. The effects of climate change are likely to be many, with some such as increased water temperatures and increased severe weather, potentially have opposing effects on phytoplankton growth. There therefore remains a great difficulty in confirming how the effects of climate change are likely to influence HABs in the UK, or indeed elsewhere. This is further compounded by our lack of understanding of whether the purported increase in HABs in recent decades is real and, if it is, what has caused it [27]. However, expected changes to UK shelf seas are reported to include increases in sea surface temperature, an increase in strength and duration of water column stability through thermal stratification, a possible increase in high wind speed and storm events, increased carbon dioxide (CO_2_) in the atmosphere leading to ocean acidification and a decrease in the pH of surface waters together with potential changes in the patterns of water mass circulation [46]. With increasing sea temperatures, changes in the balance of diatoms and dinoflagellates in UK waters might be expected. Diatoms typically dominate in cool, nutrient-rich, turbulent waters. In contrast, dinoflagellates are adapted to warm and stratified seas. Each phytoplankton species is typically adapted to grow over a range of temperatures that are characteristic of their normal habitat. Growth rates are usually higher at higher temperature, but considerably lower beyond an optimal temperature [44,47]. Temperature effects on phytoplankton growth and composition are more important in shallow coastal waters, which experience larger temperature fluctuations than oceanic waters. Predicted increasing sea surface temperatures of 2–4 °C may shift the community composition toward species adapted to warmer temperatures as observed in the temperate North Atlantic [48]. This may improve the growth success of brevetoxin-producing *Karenia* species should they be introduced. Increasing water temperature is also linked to increased strength and duration of the seasonal stratification of shelf seas. Again, this is likely to favour flagellate species.

## 3. Potential New/Emerging Brevetoxin Threats for UK Waters

To date, NSP has been confined to the Gulf of Mexico, the Atlantic coast of the United States of America (USA) and to New Zealand, with no reported occurrence in the UK or Europe [13,49]. The metabolism of *K. brevis* has been determined in cockles (*Austrovenus stutchburyi*), mussels (*Perna canaliculus*), Pacific oysters (*Crassostrea gigas*), Eastern oysters (*Crassostrea Virginia*) and clams (*Mercenaria* sp.). NSP has been associated mainly with oysters, clams, whelks, cockles and mussels [1,5,50,51].

Whilst no BTXs have been identified to date in UK waters, with the possible further global expansion of *Karenia* blooms and the potentially favourable conditions in UK waters to support the genus, these toxins could potentially become established in the UK. Although, with no reports of clinical cases or toxin occurrence in Europe, but with some trends in expansion of algal producers, the risk of these toxins appearing in European waters is at best moderate [52]. Given the significant effects resulting from blooms of NSP-producing algae in New Zealand where water temperatures are not too much higher than those in parts of the UK, NSP is a risk that should not be discounted.

## 4. Brevetoxin-Producing Phytoplankton Detection Methods

Potentially biotoxin producing phytoplankton are enumerated within a number of shellfish safety regulatory monitoring programmes that are undertaken to fulfill the requirement of EU directives that biotoxin producing phytoplankton are enumerated in shellfish producing waters. In all UK programmes eight species or genera of potentially biotoxin producing phytoplankton (*Pseudo-nitzschia* spp., *Alexandrium* spp., *Dinophysis* spp., *Prorocentrum lima*, *Prorocentrum minimum*, *Lingulodinium polyedrum* and *Protoceratium reticulatum*) are enumerated on a routine basis. Other potentially harmful species including, the fish killing *K. mikimotoi*, are recorded and reported to the regulator in the event of high density or unusual occurrence.

### 4.1. Sample Collection

Phytoplankton samples are collected from areas representative of shellfish harvesting sites. In Scottish waters, where the majority of HAB events occur, this monitoring is based on a “pod” system that may include a number of harvesting areas. Each pod containing a representative monitoring point (RMP), at which sampling occurs.

Water samples are collected using a variety of techniques. Most frequently a tube sampler (10 m) is used to allow the collection of a depth integrated sample that averages any vertical non-homogeneity in phytoplankton concentration. However, when water depth is shallow sometimes with a pole sampler or bucket is used. The frequency of collection varies being monthly in winter, bi-weekly in early spring and late autumn and weekly in the high risk summer months. A portion (500 mL) of the collected sample is fixed with the preservative Lugol’s iodine on site and transported to the testing laboratories for analysis.

### 4.2. Sample Enumeration

Standard inverted light microscope methods using the Utermöhl technique are typically the default method for cell enumeration in regulator monitoring [53], and are applied in all of the UK programmes. The method has been accredited by the United Kingdom Accreditation Service (UKAS) and is clearly fit for purpose for routine enumeration of the majority of the harmful organism that are present in UK waters (the exception being *Azadinium*, as these small cells cannot easily be discriminated by light microscopy at the speed that is required within a regulatory monitoring programme). However, the Utermöhl technique is applicable to detection of relatively large *Karenia* species. Alternative enumeration methodologies are detailed in [54] and include alternative light microscope methods incorporating a settlement bottle method and/or a range of alternative counting chambers such as the Haemocytometer, Palmer Maloney cell and Sedgewick Rafter Cell. None of these are likely to provide any additional benefits to the enumeration of brevetoxin-producing species.

Full enumeration of all HAB species is much more time demanding than monitoring only the expected HAB species in a region, particularly if toxicity is likely to occur at low cell densities, and is hence impractical financially and logistically in most monitoring programmes due to the need for rapid result reporting. While such full counts do provides a much greater likelihood of identifying new harmful species should they become present in the water column, the relative ease of identification of *Karenia* spp. and its routine reporting at elevated concentrations mean that the genus at least is unlikely to be missed by monitoring.

### 4.3. Particle Counting Methods

A range of automated counting approaches exist as an alternative to microscopy. Flow cytometry is a popular tool for the rapid identification and enumeration of different populations in mixed microbial communities [55]. Flow cytometry is most appropriate for the enumeration of small cells such as pico-plankton or bacteria Flow cytometry involves the direction a beam of laser onto a hydro-dynamically focused stream of liquid containing the cells of interests. Multi parameter discrimination and enumeration of cells is then based on the forward angle scatter (FSC) (0.5°–5°), side angle light scatter (SSC) (15°–150°) and fluorescence at a range of wavelengths. Detection and discrimination is typically based on bi-plots of light scatter, autofluorescence or laser light excitation of fluorescent stains or probes [55,56]. Dense blooms of large cells may clog the flow cell and larger cells, particularly when fixed with Lugol’s iodine, do not generate a scatter pattern that allows the genus or species level discrimination required for HAB monitoring, with molecular methods then being required [57].

Another approach involves the use of Imaging Flow Cytometry. The most high profile and commercially produced instrument in this category is the FlowCAM. Such instruments are similar to a flow cytometer but can routinely enumerate larger cells and also contain an imaging microscope allowing an image of each enumerated particle to be saved. Software allows for “training” of the instrument to assist in analysis and classification. This approach has been used successfully in the enumeration of the high biomass HAB *K. brevis* [58], so would be a potential tool for enumeration of brevetoxin-producing phytoplankton allowing more rapid enumeration of cells of interest than can be achieved by a microscopist. However there is as yet insufficient evidence to suggest that these instruments are suitable for field based monitoring programmes, with one disadvantage of the method for routine monitoring of HAB species within mixed populations being that preservation of the sample is not recommended as the loss of fluorescence. Given that preservation is almost always required during the transport of regulatory samples between collection location and analysis laboratory this would make discrimination by this method more difficult.In situ instruments such as the FlowCytobot offer a potential solution to such problems, but at noted by [59] image details are not yet always sufficiently detailed to distinguish between *Karenia* species.

### 4.4. Molecular Methods

Given the morphological similarity of different species of *Karenia* and (almost total) current absence of all but *K. mikimotoi* in UK waters, it is perhaps unlikely that routine monitoring programme microscopy or any particle counting method would discriminate an invasive *Karenia* species from the indigenous *K. mikimotoi*. Rather, such identification would require the application of molecular techniques. A range of alternative techniques for molecular identification of different *Karenia* species including nucleic acid sequence-based amplification (NASBA), fluorescent *in situ* hybridization (FISH) and microsatellite markers. The specific application of such approaches to *Karenia* are reviewed more fully by [22] with a recent publication by [60] developing the real time PCR approach.

### 4.5. Indirect Estimates of Phytoplankton Biomass

The most readily available indirect estimate of phytoplankton abundance is based on fluorescence, either though directly deployed instruments, satellite remote sensing, or through the collection of water samples that are processed in the laboratory.

Fluorescence provides an estimate of the chlorophyll content of the phytoplankton community and hence its biomass. The reliance on total chlorophyll means that the information is most valuable for high biomass organisms, with applications for example for red tides in Ariake Sound Japan [61]. Development of algorithms for the specific detection and classification of harmful species based on parameters such as absorption, total backscatter, and water-leaving radiance have had some success in identifying and tracking blooms. An example of such an approach is the remote sensing of a major *Karenia mikimotoi* bloom in Scottish waters in 2006 [23]. Work is ongoing to make such systems operational in the UK [62,63].

A similar approach is taken by the HAB forecasting system developed for *K. brevis* in the Gulf of Mexico [64]. HAB forecasts are made twice weekly during bloom events, using a combination of satellite derived image products, wind predictions and a rule-based model derived from previous observations and research. Blooms are detected and defined using ocean colour satellite images, bloom transport is then predicted using hydrographic modelling with passive particle transport. This system is now operational in the region, with the federal government making the twice-weekly forecasts and working closely with state agencies for ground verification., Satellite detection, has a range of drawbacks, including low likelihood of obtaining cloud-free images, relevance of satellite images for only surface populations and the detection of only dominant organisms [65].

Finally, the presence of a pigment based biomarker, gyroxanthin-diester within *Karenia* [66] and related genera [67,68] has led to the development of in-situ remote sensing approaches such as the Optical Plankton Detector (BreveBuster) [69] that has the potential to be deployed on underwater vehicles [70].

### 4.6. Future Monitoring of Karenia

Overall, phytoplankton species of the genera *Karenia* have been found in UK waters and the establishment of new toxic species does present a risk, albeit a relatively low one. The current regulatory biotoxin producing phytoplankton monitoring programmes are able to detect these algae to the genus level, and this microscopy based approach is clearly fit for purpose in this regard. However, microscopy is unlikely to identify *Karenia* species potentially toxic to humans were they to become present. To allow any invasive species to be discerned, the use of molecular based monitoring within water monitoring programmes is viewed as the most appropriate approach. However, once more it is unlikely that such an approach would be applied routinely in cost conscious monitoring programmes on a precautionary basis alone. Hence, its application is likely to be restricted to the species verification of high biomass blooms, or perhaps more usefully at a number of sentinel sites co-incident with major shellfisheries and/or major ports.

## 5. Toxin Testing Methods

### 5.1. Mouse Bioassays

The classical method of detecting BTX-group toxins is by the Mouse Bioassay (MBA). This provides a direct determination of total sample toxicity using live mammals. Following diethyl ether extraction, the crude lipid-extract is injected *i.p.* into mice and results are reported as Mouse Units (MU) per 100 g. It is estimated that one MU is equivalent to 4 µg BTX-2 equivalents. Several studies e.g., [19] have indicated that diethyl ether does not extract some of the BTX-group toxins effectively. The MBA does provide a measure of the overall toxicity of the sample and is relatively simple however there are disadvantages. The method gives no information on toxin profiles, there are questions over the extraction efficiency for some of the toxin group, and the bioassay is inherently variable due to specific animal characteristics. More recent studies [71] of mouse bioassays for the brevetoxins in shellfish found that oyster extracts caused pronounced matrix effects. In spite of these drawbacks some maintain their interest in the use of mouse bioassays [71] and even suggest refinement of mouse bioassays, including the use of different sample extraction solvents [72]. The general trend however is to embrace new and more sensitive chemical methods and in many countries the MBA is considered undesirable for ethical reasons. It is also worth pointing out that use of mouse bioassays, for marine toxins in general, has caused undue focus on mammalian toxicities based on intraperitoneal injection, when oral toxicity is actually the relevant risk assessment parameter [73].

### 5.2. Chemical Methods

As with any chemical method involving quantitation of specific toxins or toxin groups, methods of analysis require determination alongside certified reference materials. To date the only commercially available reference material standards are for BTX-2 and BTX-3. As a result, and possibly as a consequence of the availability issue, no analytical methods have been validated formally through interlaboratory study.

#### 5.2.1. Extraction

BTX analysis is complicated by both matrix interferences and metabolism [74]. Requirements for successful extraction of BTXs prior to analysis are highly dependent on the nature of the samples under investigation and the physico-chemical properties of the BTX toxins present. BTXs produced by algae are lipophilic compounds, but extraction of all BTXs is made more complicated by the presence of hydrophilic shellfish metabolites [75,76] and also polar metabolites from aerosols [77]. PbTx-2 also appears to rapidly and irreversibly bind to or react with shellfish tissue components, resulting in poor recoveries of the toxin from spiked shellfish homogenate [76,77].

Extraction of BTXs in seawater has been achieved through trapping toxins on hydrophobic solid phase extraction sorbents and eluting with methanol [15]. Toxins in samples of marine aerosols have been collected with glass fibre filters in air sampling devices, with toxins extracted from filters using acetone [15]. In addition to the di-ethyl ether extraction method prior to the MBA, both acetone and methanol solutions have been used for extraction of BTXs from shellfish, with the latter deemed more suitable for extraction of the more polar metabolites [18]. 80% methanol solutions have been used for successful extraction of BTXs in shellfish implicated in NSP in New Zealand e.g., [78]. Other workers have reported the use of additional clean-up steps including the removal of neutral lipids from acetone extracts of oysters with hexane and C18 SPE clean-up, plus the purification of oyster extracts with normal phase (silica) SPE to improve toxin recoveries [76].

#### 5.2.2. Conventional Chromatography Methods

Conventional chromatographic methods relying on spectroscopic detection or following functional derivatisation are not commonly reported, with HPLC and diode array UV detection mostly undertaken for fractionation of toxic extracts prior to confirmation by LC–MS [79]. These methods provide instrumental-based chemical detection of toxins, with confirmation arising from the presence of chromatographic peaks at the same retention time as analytical standards. Consequently these methods are less specific and more prone to false positive results than mass spectrometric detection methodologies. Methods have been developed for the HPLC–UV detection of BTXs on sorbent filters used to concentrate toxins present in aerosols and seawater [80]. The use of diethylaminocoumarin carbamate for the derivatisation of BTXs has been reported to facilitate HPLC-FLD detection [81]. Analysis of diethylamino-coumarin carbamate BTX-3 showed two peaks corresponding to the two expected hydroxyl substitutions, with identities confirmed by mass spectral analysis. [82] reported the use of Micellar electrokinetic capillary chromatography (MEKC) with laser-induced fluorescence (LIF) detection to measure four BTXs at trace levels. The method required pre-analysis derivatisation of BTXs with a terminal alcohol group to form highly fluorescent derivatives. The approach was applied to the analysis of PbTx-2, 3, 5 and 9 in cell cultures and fish tissue, providing excellent method detection limits of approximately 4 pg/g.

#### 5.2.3. LC–MS Methods

A major focus for development of analytical instrumentation methods has been on the use of LC–MS methodologies given the high degree of specificity they provide [83]. The technique of LC–MS involves the injection of a sample extract onto a LC column, with the separation of analytes achieved through use of appropriate column and mobile phases. Components of interest are eluted from the column into the source of the mass spectrometer, whereupon analytes of interest are ionized and detected. To date LC with electrospray mass spectrometric (LC–ES–MS) and tandem mass spectrometric (LC–MS/MS) methods have been used extensively for the identification of BTXs in algae, fish and shellfish [1,2,4,19,74,75,76,78,79,84,85,86,87,88,89,90,91,92].

With the use of LC–MS and LC–MS/MS detection methods, highly specific identification of individual BTX congeners can be successfully conducted, given the availability of appropriate analytical standards. Even without standards, published data on expected diagnostic parent and product ions could potentially be used a screening tool for BTXs in shellfish, although no performance data would be available when taking such an approach.

Several authors have described the use of LC–MS for analysis of BTX metabolites present in oysters using reverse-phase chromatography with selected ion monitoring in positive mode [75,76,77]. Good performance was reported for an LC–MS method used in a thirteen laboratory collaborative study for the analysis of oysters spiked with PbTx-3 (mean recovery = 78%) [93]. Results compared well to those generated by ELISA and the MBA. However, results were not so good for the measurement of naturally contaminated shellfish containing BTX metabolites, resulting in poor between lab variability. Full scan LC–MS has also been described with a single quadrupole detector for the determination of BTXs in water, air and shellfish samples [13]. Other workers have reported that atmospheric pressure chemical ionization performed better than electrospray ionization in the LC–MS detection of the brevetoxin-2 in seawater [94]. The use of both LC–MS/MS and accurate mass measurement for the identification of toxin metabolite structures has been demonstrated [84], with other workers describing the use of selected reaction monitoring (SRM) LC–MS/MS analysis for a range of specific BTXs [78,85]. They applied this method to the determination of toxin profiles in cockles, mussels and oysters implicated in an NSP event in New Zealand. Most recently, the technique has been used for the characterisation of BTX metabolites in hard clams exposed to blooms of *Karenia brevis*, confirming the absence of the principal algal toxins (PbTx-1 and PbTx-2) and the presence of a range of metabolites including products of oxidation, reduction, hydrolysis and amino acid/fatty acid conjunction [1]. These results returned from these methods have been found to correlate well with those determined by the NSP bioassay and the Enzyme-linked immunosorbent assay (ELISA) method.

Generation of important BTX and BTX metabolite standards is continuing [95] including the availability of six different BTXs and metabolites from Cawthron Institute in purified form [96]. It is likely that future developments will enable the formal validation of suitable LC–MS methodology for the quantitation of BTXs in shellfish. Work is still ongoing to evaluate the use of BTX biomarkers in further developing LC–MS/MS methods for monitoring BTX exposure and toxicity in shellfish [1]. In New Zealand, routine monitoring of BTXs by LC–MS is ongoing and quality control procedures have been developed including recovery and calibration slope controls [97]. In 2012 Cawthron published a single-laboratory validation of a LC–MS/MS method for six BTXs (PbTx-3, BTX-B5, S-desoxy BTX-B2, BTX-B2, PbTx-2 and BTX-B1) in four species of shellfish, including mussels, oysters and clams. Recovery and precision appeared acceptable for the majority of toxins and the method provided good levels of sensitivity (LOD and LOQ) and ruggedness to experimental deviations [98]. A low number (*n* = 4) of natural samples contaminated with BTXs prevented the thorough comparison of LC–MS/MS results against the NSP MBA, although the mean ratios between the test results were used to propose a conservative regulatory action of 0.8 mg/kg BTX-2 equivalents.

The BSX and KBT isolated and purified from *K. brevisulcata* cultures have enabled the calibration of a sensitive LC–MS/MS method for analogues of both toxin groups [99]. Method performance was validated and shown to be acceptable in terms of method linearity, sensitivity, recovery and precision. LC–MS/MS monitoring is ongoing for both types of compounds in cultures, water samples and shellfish [99].

### 5.3. Biomolecular Methods

The development of a wide range of biomolecular detection methods including both functional and biochemical assays has been shown to be applicable to a variety of sample types. Methods applied to BTXs include a cytotoxicity assay, receptor binding assay and immunoassays. Given the modes of action, some of the assays are applicable to the detection of more than one group of marine toxins e.g., [100].

#### 5.3.1. Cytotoxicity Assay

The cytotoxicity or neuroblastoma (N2A) assay is a sensitive and useful screening tool for detecting BTX-like activity (sodium channel enhancers) in extracts of shellfish [101]. It is based on the actions of BTXs, as well as ciguatoxins, on voltage gated sodium channels. The response of the assay reflects the mixtures of BTXs and has been used for examining cytotoxic fractions of shellfish extracts [76,84,102]. Neuroblastoma cells are used by some authors due to the high numbers of sodium channels in their cell membrane [103]. Alternatively, their advantages may relate more to practicalities and ethics. These are cancer cells, easily grown and maintained as secondary cultures, so there is no ongoing requirement for primary culture nerve cells, use of which requires animal sacrifice. The assay cannot distinguish between individual BTXs, and is more sensitive to less polar BTX metabolites [19]. The relative sensitivity of the assay to three separate metabolites has been found to parallel the relative sensitivity determined by the MBA [104]. Issues have been noted in the past with use of the assay for shellfish toxicity testing due high variability in an interlaboratory study, albeit involving a low number of laboratories [93]. Matrix effects and a poor correlation with the BTX MBA in contaminated oyster samples have also been described [50], with differences in assay performance remaining unexplained. The method requires long incubation times, particularly when needing to achieve high sensitivity of analysis. Given the applicability of the assay to detection of ciguatoxins (CTX) and the higher sensitivity of the assay for CTX as compared to BTXs, potential specificity issues with the assay have been noted [100]. However, in practice this is not an issue given that these two toxin groups are not found in the same organisms, *i.e.*, NSP being found mostly in shellfish as opposed to Ciguatera Fish Poisoning (CFP) in fish. Flexibility could be an advantage, as with minor modifications the assay can be utilised for testing for both sodium channel blockers (saxitoxin and tetrodotoxin) and activators (BTX and CTX) [101]. Overall, whilst the method provides a useful and sensitive tool, it was not deemed an appropriate replacement for official control monitoring in place of the MBA by some authors [93,103]. However, with further experience in the method and with routine protocols in place for passaging the assay cells there could still be potential for further developments in this area, whilst the assay certainly remains an effective research tool. New flow cytometric assays using N2As in combination with voltage sensitive dyes are more rapid than the cytotoxicity assay, and respond to brevetoxins [105] but have not been applied to detecting brevetoxins in shellfish extracts. Similarly, a new fluorescence microscopy method involving the use of an alternative cell line to N2A cells, was developed for the assessment of cytotoxicity. This has been reported as providing potential advantages over the traditional N2A assay, but is also yet to be applied to extracts of contaminated shellfish samples [106].

#### 5.3.2. Receptor Binding Assays

This is the simplest of the pharmacology-based assays which measures brevetoxins in shellfish extracts by competitive displacement of a tritium-labelled BTX (^3^H-PbTx-3) from sodium channel binding sites in isolated rat brain membranes [107] or whole cell preparations [108]. The method is a relatively simple, sensitive and rapid tool for brevetoxin analysis. Its performance has been demonstrated through an interlaboratory study on shellfish samples containing multiple BTXs, and it is considered a potential option for replacement of the MBA [93]. Whilst the method is also applicable to ciguatoxins, it has been found to be 3–24 times more sensitive to BTX analysis than for ciguatoxins [100]. The binding affinity of BTX metabolites has been investigated and found to be variable depending on the specific toxin [104], with more polar metabolites showing ten times less the affinity for the receptor as compared with PbTx-3. Membrane preparations from animal tissues are required but whilst the use of the radiolabel can be perceived as a disadvantage, the technique is becoming more popular particularly as a similar assay has become an official method of analysis for saxitoxins (AOAC Official Method 2011.27 [109]). Another noted disadvantage is the presence of matrix effects in the assay [110]. An RBA with a high throughput format using microplate scintillation technology has also been described [111] which allows parallel assay completion within 3 h.

More recently still, a competitive fluorescence-based binding assay was reported for study of the inhibition of binding at the BTX receptor in rat brain synaptosomes. The authors reported a rapid assay (<3 h), applicability to both Type-A and Type-B BTXs whilst removing the need for radiolabelled materials and potentially providing another area of research for continued developed of RBAs [112,113]. Although the authors point out that the assay has the potential advantage of having reduced background and fewer matrix problems than the radiolabel-based assay, it has not yet been demonstrated for shellfish extracts.

#### 5.3.3. Immunoassays

Immunoassays are structure-based *in vitro* methods which are found to be highly specific and sensitive detection methods [4]. They are also applicable to field monitoring scenarios and allow high throughput analyses. A competitive radioimmunoassay (RIA) was originally developed to detected PbTx-2 and PbTx-3, with a limit of detection of 1 nM [114]. A sensitive and specific radioimmunoassay was also developed by [115], specific for PbTx-2 toxins with no cross reactivity to type 1 and an improved limit of detection of 0.3 ng/mL [116]. However, these are no longer being developed due to issues relating to disposal of radioactivity [117].

Since this time, work has continued predominantly with the preparation of specific monoclonal antibody enzyme-linked immunosorbent assays (ELISA). These assays bind toxic BTXs and non-toxic derivatives with the same level of activity and work has shown that more than one antibody would be required for detecting the full range of BTXs. Whilst the antibody is highly specific to the type-B BTXs it showed low cross reactivities with type-A BTXs, it also showed low cross reactivity to other marine toxins which may have interfered with the assay [118]. It is also noted that similar immunoassay responses will be found with all other type-B toxins [107,118]. The NSP ELISA developed by the University of North Carolina Wilmington (UNCW) and described by [118], was found to provide high sensitivity (0.025 mg/kg) analysis of BTXs in shellfish, seawater and clinical specimens. It was also found to have no issues in relation to matrix interferences, even without any form of pre-treatment, dilution of purification. The collaborative study of [93] showed that this assay performed well for the detection of BTXs in contaminated oysters, with results correlating well with those from the MBA and with LC–MS [50]. This ELISA has been validated in-house and proposals have been put in place for AOAC validation by collaborative study and adoption of the method as a Type 1 National Shellfish Sanitation Program (NSSP) analytical method to replace the MBA for NSP monitoring (Proposal 07-104). However, this validation was never realised and the assay components, although commercially available (MARBIONC, Wilmington, North Carolina, USA) are not sold as a kit.

Another ELISA was developed in New Zealand incorporating anti-PbTx-2 antiserum and demonstrated excellent cross reactivities for type-B toxins in relation to PbTx-3 [119]. This was found to have a working range of 90–2100 pg PbTx-3 eq./mL and recoveries for PbTx-3 and two metabolites varying between 58% and 87%. The assay was successfully applied to the analysis of BTXs in dolphin blood. Other workers have concluded that the same ELISA provided the most sensitive bioassay for certain BTX metabolites (dihydro BTX-B and BTX-B2) but not for others (*N*-palmitoyl-BTX-B2) [104].

More recently a faster commercial NSP ELISA assay has been made available (Abraxis, Warminster, PA, USA) based on the work originally published by [119] which is a quantitative and sensitive assay applicable to both water and shellfish samples. The relatively simple and cost-effective test is also a direct competitive ELISA based on the recognition of BTX by specific antibodies, with detection of colourimetric changes in plates with a 96 well format. Whilst providing a quantitative result by quantitation against PbTx-3 standard, the manufacturers note this is a screening tool and positive results should ideally be confirmed by a suitable alternative method. The specificity of the test has been demonstrated to exclude cross reactivity with a wide range of organic and inorganic compounds. Single laboratory validation of the assay has been conducted for acceptance of the method for use in the NSSP. Toxin recovery is quoted as 86% for water and 104% for shellfish samples, with a working linear range of 0.5 to 100 ng/g in shellfish extract. The acceptable ruggedness and repeatability of the assay has also been demonstrated. The specificity of the assay is thought to be similar to that of the UNCW ELISA, being high for type-B toxins (83% to 133% relative to PbTx-3) although lower for PbTx-6 and PbTx-1 (13% and 5%) [120]. It is hoped that a multi-laboratory validation will be conducted within the next few years, incorporating the Abraxis ELISA amongst other confirmatory techniques [121], with the target of recognition by the ISSC within the next two years [122]. Both the Abraxis ELISA kit and the UNCW (MARBIONC) ELISA components were compared with an in-house mouse bioassay by [71]. One of the general conclusions of these studies is that both ELISAs appear well suited for brevetoxin screening. A note of caution relates in general to the use of commercial test kits for toxin testing, with the potential for test kits to vary between production batches and/or for the manufacturers to change performance characteristics. Such changes could potentially compromise their use for reproducible analysis of shellfish for toxin activity. Currently there are no ELISAs available containing an antibody mix that determines both BTX structural types equally well. Overall, these assays are useful as screening tools, but cannot be used for the determination of sample toxicity when unknown profiles of toxins are present. Therefore other confirmatory methods are required for full quantitation of toxicity or for the determination of BTX profiles in shellfish.

#### 5.3.4. Biosensor Methods

In 2009, researchers published work describing the application of a Surface Plasmon-Resonance detection method for PbTx-2 together with a range of other ladder-shaped polyethers including Yessotoxins (YTXs) [123]. The method uses one specific YTX (desulphated-yessotoxin; dsYTX) which is immobilised on a sensor chip. The technique involves the detection of the ability of analytes to inhibit the binding of phosphodiesterase II to the immobilised dsYTX. Detection was successful for PbTx-2 as well as YTX and dsYTX itself. Dose-response curves were generated for PbTx-2 enabling the confirmation of half inhibitory concentrations in the low µM range. Whilst demonstrating potential, there are clear specificity issues given responses from potentially many different ladder-shaped polyethers, and the technique has not been tested in samples of water, culture of shellfish [124]. A major advantage with biosensor methods is the inherent sensitivity, enabling the dilution of matrix effects which can be an issue. The equipment itself can be very expensive and probably impractical for high throughput monitoring unless used for a large number of different tests. This is heightened through the need to either buy kits from manufacturers or to develop binders and chips in-house.

Other biosensor methods reported for BTXs and potentially useful as research or screening tools [117] include one consisting of a screen printed electrode system for electrochemical immunosensor detection which enabled detection of PbTx-3 with an LOD of 1 ng/mL [125]. [126] also reported the use of a neuronal network biosensor (NNB) for the detection of PbTx-3 in both solution and diluted seawater. Although the method was found to provide good sensitivity of detection (0.296 and 0.430 ng/mL in buffer and diluted seawater respectively), there was a lack of specificity given a twenty-fold increase in method sensitivity for the detection of saxitoxin.

## 6. Suitability of Toxin Testing Methods

### 6.1. Suitability of Existing and Potential Methods for Brevetoxin Testing

Table 1 summarises the available methods applicable to the detection of brevetoxin-producing phytoplankton and shellfish brevetoxins. One attractive approach for determination of BTX sample toxicity could potentially be the application of one of the biomolecular methods described. The receptor binding assay is gaining popularity for the determination of Paralytic Shellfish Poisoning (PSP) in shellfish samples and given its status as an Official Method of Analysis following AOAC validation, the application of the BTX RBA method is a potential way forward. However, with the BTX method not yet shown to be truly effective for the full range of BTX congeners and the systems not currently in place within the UK official control testing regime, this would be a long term developmental process. Similarly the cytotoxicity and biosensor methods whilst showing great potential are not yet in a position to be considered as potential replacement methods given specificity issues and other conclusions drawn by researchers relating to their use as official control monitoring tools. They do remain potentially effective research tools.

The ELISA methods have been shown to provide a sensitive determination of type-B BTXs in particular, with good specificity in terms of a lack of cross reactivity to other marine biotoxins. Whilst the response to Type-A toxins is significantly lower, the response is still present at useful concentrations. Currently there are no ELISAs reported containing an antibody mix responding to both toxin types equally. The methods also appear to have limited effects from matrix components present in shellfish extracts, as well as seawater and clinical samples. With the assay appearing to correlate well for Type-B toxins and metabolites with results determined using LC–MS and the brevetoxin MBA in a variety of shellfish species, this could be a good candidate for potential future methods. The original ELISA was developed and validated by UNCW, although the test is not offered commercially as a complete kit. Instead, components of the kit can be purchased (MARBIONC, Wilmington, NC, USA) although microplates must coated with (supplied) antibody by the user [71]. In principle therefore, both the Abraxis ELISA and the MARBIONC ELISA could potentially be tested, validated with suitable shellfish samples and potentially implemented given the determination of appropriate performance in collaborative study at some stage in the future.

Regarding chemical methods, conventional chromatographic detection methods have been reported but there is limited published data on method performance to make these appear viable approaches for further investigations. LC–MS methods, however, provide a more promising alternative approach. The high specificity and sensitivity of the technique together with the increasing availability of some of the important BTX standards, makes this a good choice for future investigations. Given the presence of this technology at both of the official control laboratories within the UK monitoring programme, this could offer a potential applicable and cost-effective solution for future assessment and validation.

Overall it is recommended that both chemical and biomolecular methodologies should be investigated. Based on the information available in the literature at present and from discussions with leading researchers, the ELISA and LC–MS methods appear to be the most applicable for further investigation and potential development for application to official control monitoring in the UK. Depending on the scale of the testing, a first stage screen with a suitable commercial kit which has been validated for the samples of relevance, followed by confirmation by LC–MS/MS currently appears to be the most suitable option.

**Table 1:** Summary of methods applicable to the detection of brevetoxin-producing phytoplankton and shellfish brevetoxins.

Method	Advantages	Disadvantages
Microscopy	Detection of *Karenia* genus	Detection not species-specific
	Fulfils requirement of legislation	No evidence for shellfish toxicity
Particle counting methods	Detection of *Karenia* genus	Detection may be compromised when analysing dense blooms
	Potentially a more rapid enumeration of cells	Little evidence for suitability for field based monitoring
	Potential for in-situ analysis	Sample preservation compromises detection
		Detection not species-specific
Molecular techniques	Enables identification of different *Karenia* species	Methods still under development, with no reports of application to official testing to date
	Toxic species can be identified for verification purposes	Requires expensive instrumentation and highly trained analysts
Mouse bioassay (MBA)	Primary tool for toxicity assessment	Inability to detect all BTXs
	History of use and prevention of sickness	Ethical issues
	Relatively simple technology	Variable performance
		Not validated
Cytotoxicity assay	Sensitive functional assay	Matrix effects, high variability
	Use of cultured *vs.* primary cells	Poor correlation with MBA
	Used to detect all analogues	Noting limited data on performance characteristics of method
		Time consuming
Receptor binding assays (RBA)	Simple, sensitive, rapid	Variable affinity for BTX metabolites
	Good performance in collaborative study	Requirement for animal tissues and radiolabel
	Promising fluorescence-based binding assay	Matrix effects
		Limited development to date with fluorescence-based binding assay
Immunoassays	Specific for type-B and sensitive	Lower cross reactivity for type-A BTXs
	High throughout, fast turnaround and “in the field”	Screening tool only—no toxicity or profile data provided
	Low matrix effects	Valuable quantities of toxin required to produce antibodies
	Good correlation with MBA and LC–MS	Potential issues with commercial kits, with manufacturers changing properties or performance characteristics
	Good single lab validation and multi-lab study anticipated	
Conventional chromatography	Use of MEKC-LIF, LC-UV and LC-FLD reported	Very limited data available for determination of low numbers of toxins
	Some degree of specificity	Lack of standards and equipment
		Proof of concept required for all appropriate toxins
LC–MS (MS)	Highly specific	Expensive instrumentation
	Sensitive	Lack of all suitable standards
	Single laboratory validation performed	
Biosensor methods	Useful research screening tools	Lack of specificity
	High sensitivity	Expensive instrumentation for biosensors
	Matrix effects can be diluted	

### 6.2. Identification of Knowledge Gaps Which Might be Addressed through Further Research or Method Development

The knowledge gaps relating to research requirements for prevalence and detection of BTXs in shellfish are currently wide. These include:

- Understanding and identification of additional algal species which produce brevetoxins, in particular those found to grow well in water conditions relevant to the UK at present or in the future;
- Analysis of algal cultures by suitable methods for assessment of presence of BTXs in water samples;
- Identified shellfish species that accumulate BTXs, together with associated accumulation and depuration rates;
- The determination of BTX metabolites by LC-MS profile studies in relevant bivalve species;
- Continued evaluation of MBA-replacement methods, in particular including ELISA and LC–MS/MS;
- Understanding of BTX and BTX metabolites toxicity in relation to human exposure, including long term assessment of intoxicated people to determine potential long term affects.

From the above and in the context of risks to UK waters and shellfish, the first stage would be the identification whether the blooms with potential for BTX production occur in the UK. The next step would be the development and application of methods for the identification of toxins in harvested phytoplankton. Knowledge regarding the producers and parent toxins will then enable studies to be conducted on the uptake of these toxins into shellfish species of relevance to the UK shellfish industry. This could include both the sampling and analysis of naturally-contaminated shellfish from areas of algal blooms and/or laboratory uptake and depuration studies on shellfish fed with mass cultured phytoplankton species. With suitable contaminated materials, methodologies would need to be assessed and developed in relation to UK shellfish matrices. Further confirmatory studies would need to be conducted to assess patterns of metabolism in UK shellfish, thereby determining the most prevalent toxins accumulating in shellfish flesh and the most suitable laboratory protocols for determining and quantifying their presence. Ultimately any such methodologies would need to be formally validated to determine method performance characteristics in relation to UK shellfish species.

### 6.3. Global Regimes

Throughout the EU there have been very few instances of threats from BTX producing algae highlighted in the literature, at conferences or in working group meetings. At a recent EURL symposium on new or emerging toxin, no references were made or presentations given relating to BTX methods or issues. Similarly at a recent EURL working group on emerging toxins, BTXs were not discussed, with the focus mainly on other threats already identified in EU waters. However there is some opinion that the threat currently experienced in New Zealand, the east cost of the USA and the Gulf of Mexico may expand in future years, potentially affecting the EU.

New Zealand scientists have been actively involved for some time in both the production of standards and the development of methodologies for testing of BTXs, amongst other things incorporating BTX standards into their quality-controlled routine LC–MS/MS method for shellfish monitoring. A single laboratory validation has been published [98] although the toxins have only been seen once in recent years. Work progressing in recent years at the University of Vigo in Spain has shown some recovery issues for PbTx analogues (e.g., PbTx-2), which resulted in the need for solid phase immunoaffinity extraction clean-up prior to reverse-phase LC–MS/MS quantitation. With this approach a highly reproducible and linear method with good toxin recovery has been demonstrated [127].

In the USA, FDA scientists have maintained a program of work to identify the best options for monitoring BTXs, including both the ELISA and LC–MS confirmatory methods. The work has also been expanded to pursue the identification of BTX exposure biomarkers and toxicity in bivalve shellfish [128]. FDA Scientists are currently leading studies to validate both ELISA and LC–MS/MS methods, with current results supporting ELISA as a screening method and LC–MS as a confirmatory method [121]. The organisation have not found the limited cross reactivity to Type-A BTXs to be a significant problem, given the dominance of Type-B BTXs in shellfish as determined by LC–MS metabolite profiling studies. The examination of both oyster and clam tissues naturally contaminated with BTXs during *K. brevis* blooms by both methods and showing good correlation between the assays [1,50] supports these decisions.

Overall, it appears that the preferred global approach to future monitoring programmes is the application of both ELISA and LC–MS/MS methods for the detection and quantitation of BTXs and BTX biomarkers in shellfish. This may change if any of the other biomolecular research tools become assessed in greater depth and are formally validated. At present, there has been no feedback that this is likely to happen in the near future.

## 7. Conclusions: Proposed Options for Routine Monitoring of Phytoplankton and Toxins to Meet Legal Requirements

The current regulatory biotoxin producing phytoplankton monitoring programmes are able to detect these algae to the genus level, and are therefore fit for monitoring purposes. However, microscopy is unlikely to identify species potentially toxic to humans were they to become present. A molecular based monitoring approach would allow this but only on a precautionary basis for species verification of high biomass blooms, and/or at a number of sentinel sites co-incident with major shellfisheries and/or major ports.

Currently there are no regulatory limits for BTXs in shellfish or fish in Europe. In other parts of the world including the USA, New Zealand and Australia, maximum permitted levels have in practice been set at 20 mouse units (MUs)/100 g shellfish flesh. Expressed in BTX-2 (PbTx-2) equivalents this is equivalent to 0.8 BTX-2 eq/kg. Without regulatory limits set in EU legislation, one potential approach would be to adopt the regulations utilised in those regions currently conducting active monitoring for BTXs. Based on the evidence gathered during this review, an effective approach for routine monitoring is likely to include two separate steps. The first would be the screening of shellfish samples using a suitable assay, such as the ELISA. Secondly, screen-positive samples would be assessed using a suitable quantitative confirmation assay. Removing the MBA as an option due to ethical considerations, the strongest recommendation is for application of a confirmatory LC–MS/MS method for the quantitation of BTXs in samples determined as positive by the screening tools employed. Given that the likelihood of brevetoxin occurrence is low, this two-step approach could initially be applied as a precautionary measure at a number of selected sites. However, with full availability of appropriate reference materials, quantitative or semi-quantitative LC–MS/MS could be employed as the sole monitoring tool for a larger number of samples in tandem with routine monitoring of lipophilic marine toxins, without the need for an additional screening step.

In order to meet legal requirements associated with official control monitoring of bivalve molluscs, each of these assays would need to undergo a series of validation studies to determine full performance characteristics of the method. In addition the performance would need to be demonstrated as being able to provide at least the same level of effectiveness as the MBA. With a full collaborative study likely in the near future for both the ELISA and LC–MS/MS methods, a suitable level of interlaboratory validation will hopefully be in place to enable implementation of the method, providing there is evidence for acceptable method performance in UK shellfish demonstrated through additional single laboratory validation studies. Any such developments would need to be reviewed and the stakeholders consulted as deemed appropriate by the UK competent authority.
